# Fabrication of Cu/Al/Cu Laminated Composites Reinforced with Graphene by Hot Pressing and Evaluation of Their Electrical Conductivity

**DOI:** 10.3390/ma16020622

**Published:** 2023-01-09

**Authors:** Hang Zheng, Ruixiang Zhang, Qin Xu, Xiangqing Kong, Wanting Sun, Ying Fu, Muhong Wu, Kaihui Liu

**Affiliations:** 1 Songshan Lake Material Laboratory, Dongguan 523808, China; 2 College of Mechanical and Electrical Engineering, Henan University of Technology, Zhengzhou 450001, China; 3 State Key Laboratory for Mesoscopic Physics, Peking University, Beijing 100871, China

**Keywords:** electrical conductivity, graphene, copper and aluminum composites, hot pressing, interface

## Abstract

Metal laminated composites are widely used in industrial and commercial applications due to their excellent overall performance. In this study, the copper/graphene-aluminum-copper/graphene (Cu/Gr-Al-Cu/Gr) laminated composites were prepared by ingenious hot pressing design. Raman, optical microscope (OM), scanning electron microscope (SEM), van der Pauw (vdP), and X-Ray Diffractometer (XRD) were used to investigate the graphene status, interface bonding, diffusion layer thickness, electrical conductivity, Miller indices and secondary phases, respectively. The results demonstrate that the Cu-Al interfaces in the Cu/Gr-Al-Cu/Gr composites were free of pores, cracks and other defects and bonded well. The number of graphene layers was varied by regulating the thickness of the Cu/Gr layer, with the Cu/Gr foils fabricated by chemical vapor deposition (CVD). The electrical conductivity of the composite was significantly improved by the induced high-quality interfaces Cu/Gr structure. The increased number of graphene layers is beneficial for enhancing the electrical conductivity of the Cu/Gr-Al-Cu/Gr composite, and the highest conductivity improved by 20.5% compared to that of raw Al.

## 1. Introduction

Metal laminated composites are comprised of at least two physical or chemical components. Their superior performance is given by the achievement of enhanced combinations of properties, which could hardly be achieved for single-phase materials. The composite materials have excellent properties due to the composite matrix, which is difficult to achieve for single-phase materials [1]. Different application scenarios have given rise to different metal composites, for example, Al/Mg [2], Cu/Ni [3], TiO_2_/Cu [4], Al/Ni [5], Al/Sn [6], Cu/Zr [7], Al/Ti/Mg [8], Cu/Al [9,10,11,12], Cu/Al/Cu [13], etc. As a typical metal laminated composite, Cu/Al composite has been widely used in electric power, heat transmission, rail transit, and other fields due to the characteristics of high electrical conductivity and high thermal conductivity of Cu and the light weight and low cost of Al, which have attracted extensive attention [14]. Rimma et al. [15] achieved the combination of Cu powder and Al powder by four reciprocal extrusion passes at 400 °C. The electrical conductivity of Cu/Al composite increases with the increase of Cu content. Kocich et al. [16] used rotary swaging technology to produce Cu/Al clad composite wires with a diameter of 5 mm at 250 °C, ensuring a high strength while possessing good electrical conductivity. Han et al. [17] investigated the effect of Al/Cu diffusion bonding on the evolution of the interface at an isothermal temperature of 550 °C. The results showed that under the protection of vacuum and argon, the bonding time was increased from 15 to 25 min, and the intermetallic interactions such as Cu_9_Al_4_, CuAl, and CuAl_2_ were generated in the interface region. Hu et al. [18] found that the thickness of the secondary phases in the Cu/Al composite increased from 25 µm to 300 µm, and the electrical conductivity decreased from an initial 5.29 × 10^5^ S/cm to 3.83 × 10^5^ S/cm. In recent years, with the development of the electronic information industry, automobile lightweight technology, the national defense industry and the transformation of consumer demand to high-end, high-performance and lightweight, higher requirements have been put forward for the performance of Cu/Al composites.

With the rapid development of nanotechnology, nanomaterials, as a new type of admixture, have a high specific surface area and high activity, providing new opportunities for the development of metal laminated composites. Indeed, numerous studies have demonstrated that introducing some emerging carbon nanomaterials, such as carbon nanotubes [19], graphene-oxide [20] and graphene [21], etc., into the metal composites endows metal composites with properties that cannot be obtained in their various components. Taking graphene as an example, it is reported that graphene is a hexagonal two-dimensional lattice nanomaterial (GNPs) composed of carbon atoms, and the thickness of a single layer is only 0.34 nm. Graphene has good functional properties with thermal conductivity up to 5000 W·m^−1^·K^−1^, and experimental carrier mobility up to 350,000 cm^2^/(V·s), making it the thinnest, strongest, toughest, and best heat and conductive nanomaterial ever discovered [22,23]. Graphene has been known as an excellent reinforcement of metal matrix composites due to its excellent comprehensive properties. At present, a series of advancements have been achieved in the research of graphene-reinforced Cu matrix composites. Yu et al. [24] prepared copper graphene composites by electrodeposition; the graphene concentration in the electrolyte increased from 0 to 0.1 g/L, leading to the sample conductivity increasing from 88.3% IACS to 91.3% IACS. Dong et al. [25] found that graphene doping in W_70_Cu_30_ from 0 to 0.5 wt % increased the electrical conductivity from 42% IACS to 46% IACS. Cao et al. [26] prepared a laminar structure of Gr/Cu composites with an electrical conductivity of 93.8 to 97.1% IACS, which showed that the introduction of layered graphene can obtain excellent electrical conductivity due to the layered structure through the 2D catalytic growth of GR maintaining the electrical conductivity and thus the desired carrier transport conditions to maximize its performance. Chen et al. [27] prepared bulk Cu/Gr nanocomposites by an accumulative roll-compositing process in which graphite foil was sandwiched in a copper strip and subjected to high-cycle accumulative roll-bonding treatment, followed by hot rolling. The composites had superior strength, ductility and electrical conductivity. The electrical conductivity was above 70% IACS.

Previous research has suggested that adding graphene as reinforcement phase to Cu substrates can significantly improve the electrical conductivity of composites. However, the existing studies on graphene-reinforced Cu matrix composites mostly focus on the single Cu metal. There are insufficient studies on graphene-reinforced Cu/Al composites, and the related interface and electrical coupling response still need to be further elucidated. In addition, in order to obtain new materials with lightweight and high conductivity, graphene reinforced Cu/Al/Cu composites are worthy of attention. To address this issue, a lightweight, inexpensive, and highly conductive laminated composite material is developed in this paper. The innovative proposal is graphene-reinforced Cu in composites with Al. The graphene states, interface bonding, diffusion layer thickness, electrical conductivity, Miller indices and phase analysis of the novel graphene-reinforced Cu/Al/Cu laminated composites are investigated by using Raman, optical microscope (OM), scanning electron microscope (SEM), van der Pauw (vdP), and X-Ray Diffractometer (XRD). The contribution of graphene to the electrical conductivity of Cu/Al/Cu laminated composite in this process is demonstrated.

## 2. Materials and Methods

### 2.1. Materials

The experimental samples of commercial Cu foil (25 µm thick, 99.8%) were purchased from the Sichuan Oriental Stars Trading Co., Ltd. (Chengdu, China). The Al sheets (3 mm thick, 99.9%, 40 mm diameter) were purchased from Kierui Metal Materials Co. (Xingtai, China)

### 2.2. Methods

#### 2.2.1. Methods of Sample Preparation

**Figure 1:**
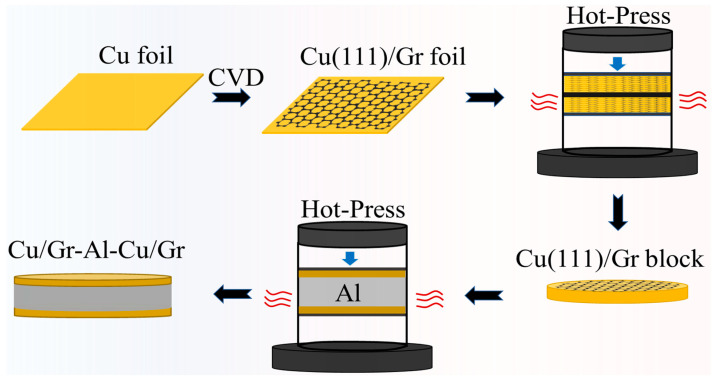
Schematic diagram of the Cu/Gr-Al-Cu/Gr preparation method.

The experimental samples were prepared as shown in Figure 1. The commercial Cu foil was placed on a quartz substrate, loaded into a chemical vapor deposition (CVD) furnace, and annealed at 1030 °C in an atmosphere of 500 sccm argon and 10 sccm hydrogens to obtain single crystal Cu(111). Methane (1–5 sccm) was then passed through. The growth was continued with argon and hydrogen until the end of cooling [21]. Using a tool, the Cu(111)/Gr foil was cut into round foils of 40 mm in diameter. The corresponding amount of Cu(111)/Gr foils were sintered in a vacuum hot pressing furnace at 10 °C/min, 900 °C and 50 MPa for 1 h to obtain a round Cu(111)/Gr block of 0.5 mm and 0.6 mm thickness. The oxide film was removed from the surface of the industrial Al sheet with sandpaper, and the surface stains of the Cu and Al sheets were removed by ultrasound. The samples were placed into the mold in the order of Cu(111)/Gr, Al, and Cu(111)/Gr for vacuum hot pressing sintering with a heating rate of 10 °C/min, a hot pressing temperature of 530 °C, a pressure of 10 MPa, and a hot pressing sintering time of 1 h. The final Cu/Gr-Al-Cu/Gr laminated composite specimens were obtained.

#### 2.2.2. Characterization

The sample was thrown longitudinally and polished by grinding. The X-Ray Diffractometer (XRD, Miniflex 600, Tokyo, Japan) with JADE 5 software was used to analyze the Cu foil Miller indices and diffusion layer phase species. The integrity of the graphene grown on the copper foil was characterized by Raman (Alpha300R, Ulm, Germany). The state of the interface and the diffusion layer were investigated, using an optical microscope (OM, BX53M, Tokyo, Japan) and a scanning electron microscope (SEM, Regulus 8100, Tokyo, Japan) equipped with energy dispersive spectroscopy (EDS).

**Figure 2:**
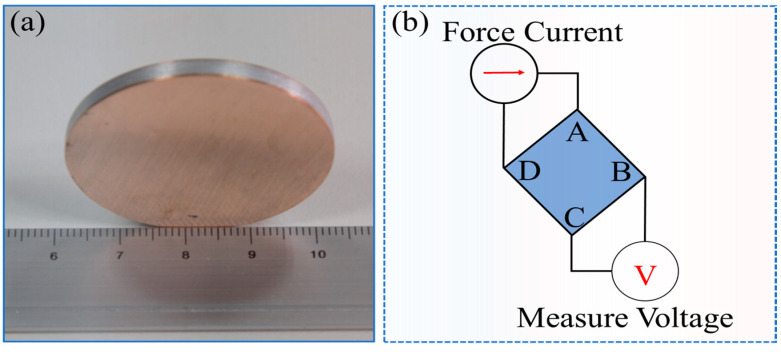
Schematic diagram of the sample for testing electrical conductivity. (**a**) Macroscopic photograph of the sample; (**b**) Schematic diagram of the van der Pauw method for measuring resistance.

The experimental specimens are shown in Figure 2a. The hot pressing samples were cleaned and smoothed by 1200 grit sandpaper and tested for conductivity by the van der Pauw method (vdP, Keithley 2182A, Cleveland, OH, USA). The principle is illustrated in Figure 2b. This measurement method was used on a small, flat-shaped sample with four terminals and a uniform thickness. A current is applied to the sample through two terminals and the voltage drop is measured through the opposite two terminals.

## 3. Results and Discussion

### 3.1. Cu Miller Indices and Graphene State

**Figure 3:**
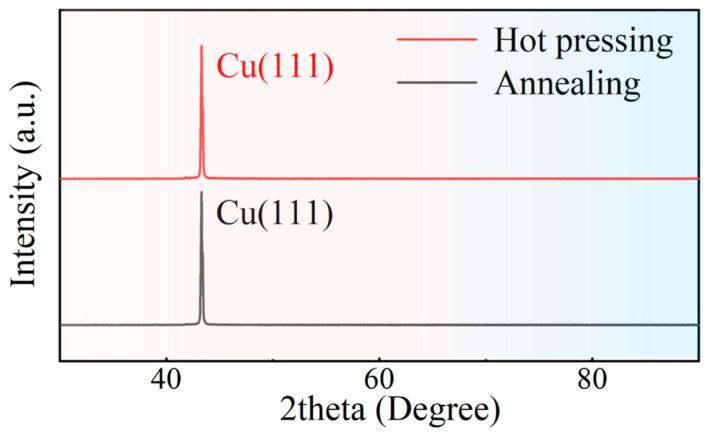
XRD tested the Miller indices of copper foil after annealing and copper block after hot pressing.

Figure 3 shows the XRD result of the Miller indices of copper foil after annealing and copper block after hot pressing. The main peak appears at 2*θ* approximately 42°. It can be clearly seen that the Cu block remained in the single crystal state after being twice hot pressed. Cu(111) has the lowest surface energy of 1.387 J/m^2^ calculated by the first natural principle [21], which results in the lowest Cu(111) formation energy and the easiest nucleation to Cu(111) when the crystal reaches the recrystallization temperature. A sample is obtained of Cu(111) foil and Al after two hot pressings. Due to the lowest Cu(111) Miller indices formation energy and the low temperature of the sample after two hot pressings, there was not enough energy to nucleate Cu(111) to other surfaces, resulting in the Cu sample remaining in a single crystal state. The presence of grain boundaries in polycrystals can lead to electron scattering, which affects the electrical conductivity of Cu, whereas single crystal Cu has only one grain without electron scattering at the grain boundaries, which helps the electrical properties of the sample. It can be seen from the figure that the Miller indices of the Cu foil do not change before or after hot pressing, ensuring a single crystal on the Cu(111) surface.

**Figure 4:**
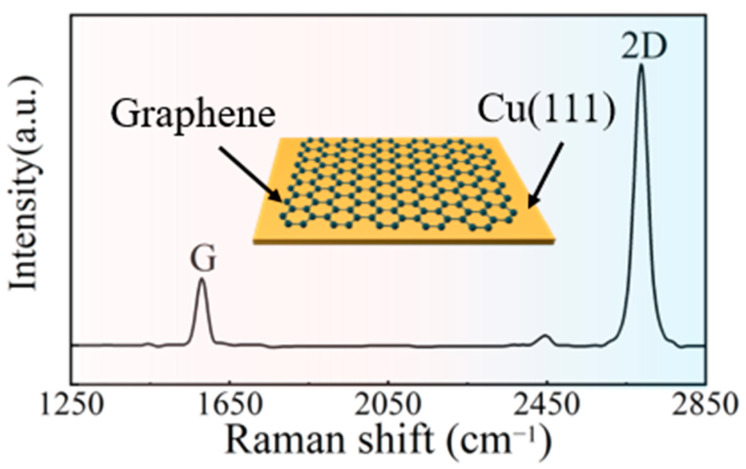
Raman characterization of graphene mapping and (inset) constructional view of Cu(111)/Gr foil grown by CVD.

Figure 4 shows the Raman characterization of graphene mapping with a G peak at approximately 1580 cm^−1^ and a 2D peak at approximately 2700 cm^−1^ [28]. The Raman spectrum of graphene consists of several peaks, mainly G peak and 2D peak. The G peak is caused by the in-plane vibration of sp^2^ carbon atoms and reflects the number of layers of graphene. The 2D peak is a double phonon resonance second-order Raman peak; it is used to characterize the interlayer stacking of carbon atoms in the graphene sample, and the intensity of the peak is also related to the laser power. This Raman graph reflects the growth of an intact layer of graphene on the surface of the Cu foil, which will bring out the high carrier mobility property of graphene.

### 3.2. Microstructure of the Composite Interface

**Figure 5:**
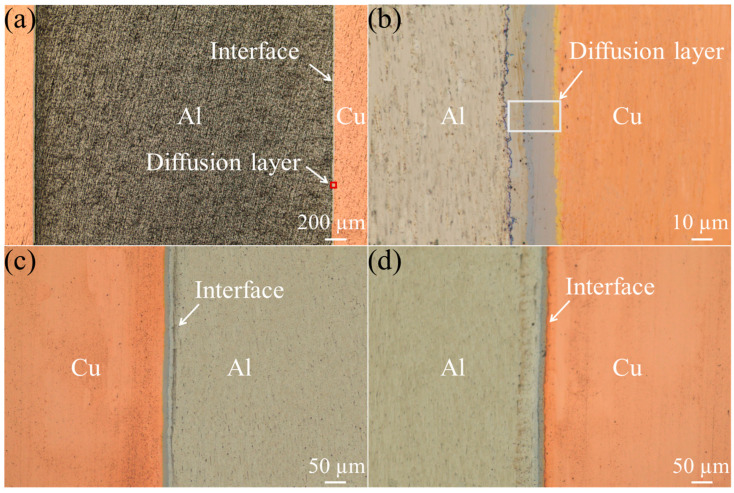
The bonding interface and diffusion layer condition of the composites. (**a**) Microscopic drawing of the sample. (**b**) Morphology of Cu/Al diffusion layer. (**c**,**d**) Microscopic views of Cu/Al upper and lower interfaces.

The bonding interface and diffusion layer condition of the graphene reinforced Cu/Al/Cu (1 mm thickness Cu(111)/Gr) composites were observed, as shown in Figure 5. Figure 5a shows that the Cu/Al bond is free of pores and cracks, and Cu/Al forms a good metallurgical bond. Figure 5b shows the morphology of the Cu/Gr-Al-Cu/Gr diffusion layer. It can be seen that four different colors appeared between the Cu/Al diffusion layers. Figure 5c,d depict the microscopic views of the interface of Cu/Al top and bottom bonding. Figure 5 clearly confirmed that the interface bonding was tight and could form a metallurgical bond.

**Figure 6:**
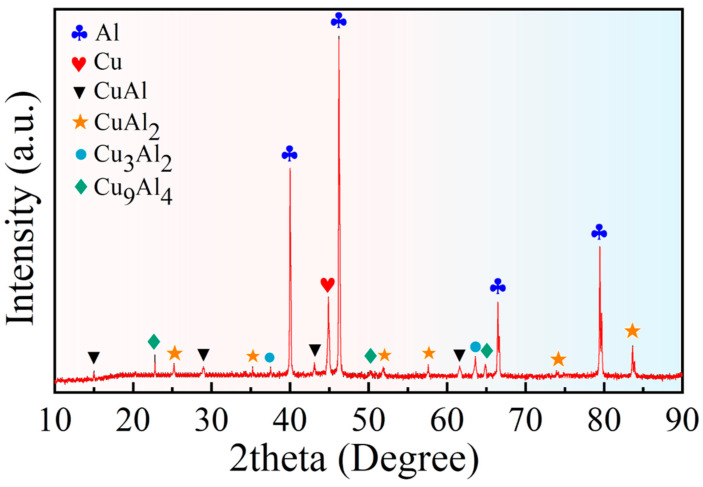
XRD test of Cu/Gr-Al-Cu/Gr laminated composites cross section.

Figure 6 shows the XRD analysis results of the Cu/Gr-Al-Cu/Gr laminated composites sample cross section. It can be found that four different intermetallic phases, i.e., CuAl_2_ (2*θ* ≈ 25°, 36°, etc.), CuAl (2*θ* ≈ 15°, 29°, etc.), Cu_3_Al_2_ (2*θ* ≈ 37°, 63°), and Cu_9_Al_4_ (2*θ* ≈ 23°, 66°) are obtained. Combined with the XRD analysis it can be seen that the four different color diffusion layers in Figure 5b are four different phases.

**Figure 7:**
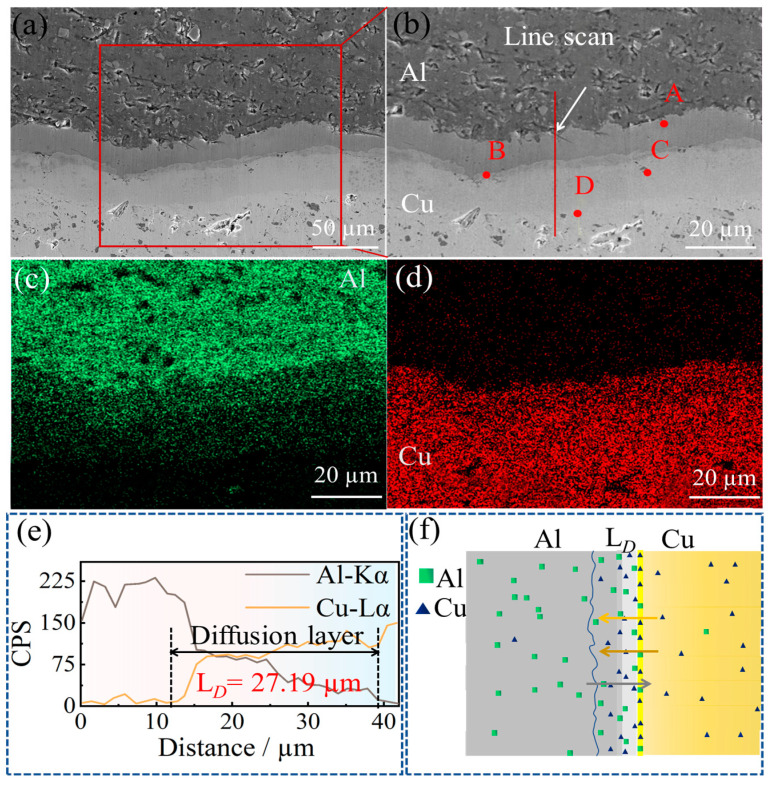
(**a**) SEM images of diffusion layer morphology; (**b**) SEM image of the sample surface scanning area and A, B, C, D are point scanning areas; (**c**) Distribution of Al elements; (**d**) Distribution of Cu elements; (**e**) Sample line scan area and energy spectrum; (**f**) Cu and Al diffusion diagram.

Figure 7a shows the secondary electron images of the diffusion layer under the SEM of the Cu/Gr-Al-Cu/Gr laminated composites, and the obvious delamination at the diffusion layer is also found by SEM. Figure 7b shows the surface scanning area of the sample. The distribution mapping of Cu and Al elements can be found in Figure 7c,d, and the area with a large overlap of Cu and Al elements is the diffusion layer. This area was quantified by line scan for a total Cu/Al diffusion layer thickness of 27.19 µm. Figure 7e shows the results for the line scan area in Figure 7b. The line scan results also easily reveal the diffusion reaction between Cu and Al. The diffusion coefficient of Cu and Al can be expressed by Arrhenius, which can be calculated from Equation (1) [29].

(1) $$D=D_{0}e^{\left(-Q/RT\right)}$$

where *D* is the diffusion coefficient, *D_0_* is the diffusion constants, *Q* is the diffusion activation energy, *R* is the gas constant, and *T* is the thermodynamic temperature. The diffusion coefficient of Cu in Al is 4.9 × 10^−16^ m^2^·s^−1^, while that of Al in Cu is 3.76 × 10^−19^ m^2^·s^−1^, at 530 °C. The diffusion coefficient of Cu atoms in Al is much larger than that of Al atoms in Cu. Cu is regarded as the first limiting element. Therefore, the formation of the Cu/Al interface reaction layer is mainly through the diffusion of Cu atoms to the Al side. Moreover, the formation energies of CuAl_2_ and Cu_9_Al_4_ are 0.78 eV and 0.83 eV. It is inferred that CuAl_2_ forms first and then forms Cu_9_Al_4_. CuAl and Cu_3_Al_2_ are exhibited to form after the formation of the previous two phases [30]. The Cu/Al intermediate phase has the characteristics of high strength, low ductility and high resistance, but the generation of the Cu/Al intermetallics can bind the samples tightly and form a good interface to improve the force and deformation of the composite. Figure 7f shows a schematic diagram of the Cu/Al diffusion reaction.

**Figure 8:**
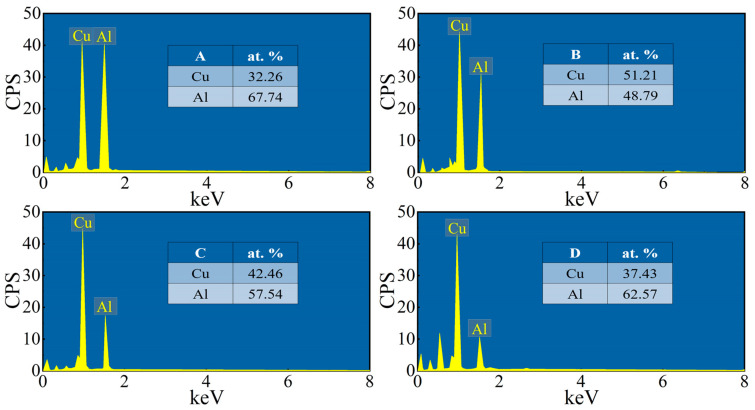
EDS result of point A-D in Figure 7b.

Figure 8 shows the EDS of points A–D in Figure 7b. Point A is close to the aluminum side, which can be identified as the CuAl_2_ phase by combining the XRD and EDS results of point A. Point B is closer to the copper side than point A, and the CuAl phase can be determined from the XRD and EDS results. The EDS results and XRD analysis at point C confirm that this is the Cu_3_Al_2_ phase. Point D is close to the copper side, and the EDS results and XRD analysis determine that this is the Cu_9_Al_4_ phase. Therefore, the intermediate phases from the Al side to the Cu side are CuAl_2,_ CuAl, Cu_3_Al_2_, and Cu_9_Al_4_ in that order_._

### 3.3. Electrical Conductivity

The vdP method of resistance measurement is repeated eight times around the edge of the sample, and the eight sets of voltages (*U*) and test currents (*I*) obtained are used to calculate the resistivity *ρ*, which can be calculated from Equations (2)–(4).

(2) $$\rho =\frac{\pi \cdot d}{\ln2}\times \left(\frac{U_{CD}}{I_{AB}}+\frac{U_{DA}}{I_{BC}}\right)\times \frac{1}{2}\times f$$

(3) $$f\approx 1-{\left(\frac{R_{AB,CD}-R_{BC,DA}}{R_{AB,CD}+R_{BC,DA}}\right)}^{2}\frac{\ln2}{2}-{\left(\frac{R_{AB,CD}-R_{BC,DA}}{R_{AB,CD}+R_{BC,DA}}\right)}^{4}$$

(4) $$R_{AB,CD}=\frac{U_{CD}}{I_{AB}};\;R_{BC,DA}=\frac{U_{DA}}{I_{BC}}$$

where *d* is the sample thickness, *f* is the vdP factor [31], *U_CD_* and *U_DA_* are the measured voltages, *I_AB_* and *I_BC_* are the measured currents, and *R_AB,CD_* and *R_BC,DA_* are the resistance of the sample.

**Figure 9:**
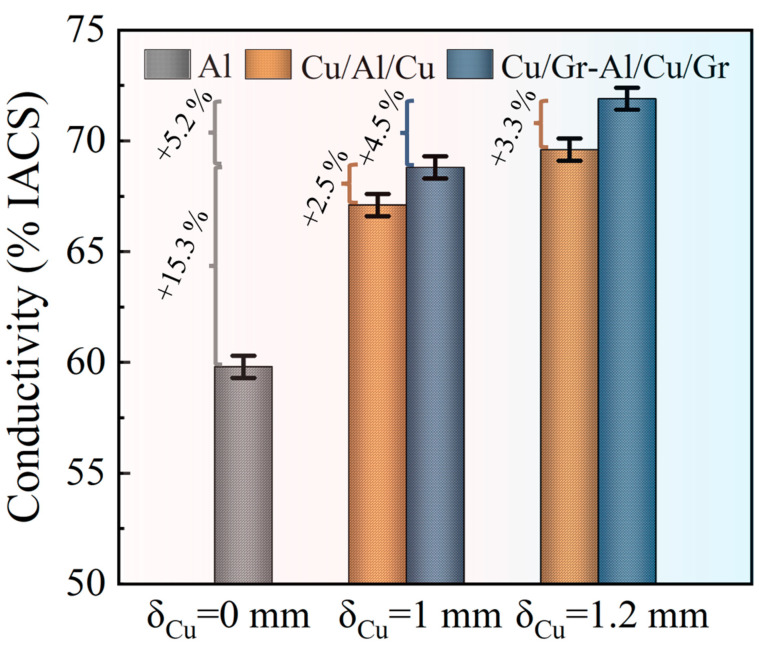
Comparison of the electrical conductivity of three different Cu(111) and Cu(111)/Gr thickness (0 mm, 1 mm and 1.2 mm) in the composites.

The electrical conductivity of the sample with different Cu(111) and Cu(111)/Gr thicknesses are shown in Figure 9. This work considered three different Cu(111) and Cu(111)/Gr thicknesses, i.e., 0 mm (Al), 1 mm (Cu/Al/Cu and Cu/Gr-Al-Cu/Gr), and 1.2 mm (Cu/Al/Cu and Cu/Gr-Al-Cu/Gr). It should be noted that as the thickness of Cu changes, the number of graphene layers changes. It can be seen that for the sample with a layer thickness of 1 mm, the electrical conductivity reached 68.8% IACS, which is 2.5% higher than the 67.2% IACS of the same sized sample without graphene. Meanwhile, for the sample with a layer thickness of 1.2 mm, the electrical conductivity is 71.9% IACS, which is 3.3% higher than that of Cu/Al/Cu without graphene (69.6% IACS). In addition, it is known that the number of graphene layers introduced by increasing the thickness of the Cu(111)/Gr layer also increased, and it was found that the sample’s electrical conductivity improved considerably after graphene was added. For example, by comparing the sample of Cu/Gr-Al-Cu/Gr with the thickness 1mm and 1.2mm, the electrical conductivity is increased by approximately 4.5% from 68.8% IACS to 71.9% IACS, whereas for the Cu/Al/Cu samples without Gr, the electrical conductivity is increased by approximately 3.6% with thicknesses increased from 1 mm to 1.2 mm. Furthermore, it can also be seen from Figure 9 that the graphene-embedded Cu/Al composite exhibited high carrier mobility from controlled experiments, and the resulting samples showed a maximum enhancement of 20.5% over the raw material Al.

The high quality graphene is grown on Cu foil by CVD [32]. Moreover, the weak adhesion energy of graphene to Cu can be improved by the hot pressing process so that graphene and Cu can form a strong mechanical bond and Cu(111)/Gr blocks without inclusions and voids can be obtained [33,34]. Moreover, graphene and Cu(111) have the same triple symmetry and very similar lattice constants, which allows graphene to be grown more completely on Cu(111), thus exploiting the high carrier migration properties of graphene [21]. In many studies on the electrical properties of graphene, there is a trade-off between electron mobility and electron density when suspended graphene or graphene interacts with a well-designed substrate; the result is a less than high conductivity of graphene-reinforced metals. In contrast, graphene materials embedded with Cu metal achieve high electron density and maintain high electron mobility. In addition, the thickness of the diffusion layer of Cu and Al is opposite to the conductivity; the electrical conductivity decreases with the increase of the thickness of the diffusion layer. The thickness of the diffusion layer of the Cu/Al composite prepared by the hot pressing method is generally smaller than that of the casting method, which also improves the electrical properties of the Cu/Al composite [18]. Therefore, by hot pressing composites Cu(111)/Gr with Al, the resulting samples will have the combined properties of lightweight and high electrical conductivity.

## 4. Conclusions

In this study, an innovative Cu/Gr-Al-Cu/Gr laminated composite was prepared by the hot pressing method, and the graphene states, interface bonding, diffusion layer thickness, electrical conductivity, Miller indices and phase analysis of the composites were studied. The main conclusions are as follows:

1. The Cu(111)/Gr sample obtained by CVD was vacuum pressed twice at 900 °C and 530 °C. The Miller indices of the sample remained at the (111) crystal face, and the Cu block was still in the single crystal state;
2. The Cu/Gr-Al-Cu/Gr laminated composites were successfully prepared by hot pressing for 1 h at a temperature of 530 °C and a pressure of 10 MPa at a heating rate of 10 °C/min. It was found that the laminated composites were well bonded without pores or cracks, there was an obvious diffusion layer at the interface bond, and the transition layer generated by the diffusion reaction of Cu and Al connected the composites. The total thickness of the diffusion layer was found to be 27.19 µm by EDS spotting, line scan, surface scan, and the intermediate phases from the Al side to the Cu side are CuAl_2,_ CuAl, Cu_3_Al_2_, and Cu_9_Al_4_, in that order;
3. The Cu/Gr-Al-Cu/Gr laminated composites prepared by the hot pressing method were able to exploit the high carrier mobility of graphene to improve the electrical conductivity of the composites and the thickness of the Cu(111)/Gr layer from 1 mm to 1.2 mm; the electrical conductivity of the Cu/Gr-Al-Cu/Gr increased by 4.5%, while the increase in the thickness of the Cu(111) layer was from 1 mm to 1.2 mm, and the Cu/Al/Cu conductivity increased by only 3.6%.

## Data Availability

All relevant data generated by the authors or analyzed during the study are included within the paper.
